# Supplementary material for: Modelling Skylarks (Alauda arvensis) to Predict Impacts of Changes in Land Management and Policy: Development and Testing of an Agent-Based Model
Source: PLoS One. 2013 Jun 6;8(6):e65803. doi: 10.1371/journal.pone.0065803 (PMC3675089; doi:10.1371/journal.pone.0065803)
Supplement: Supporting Information S4 — The skylark ODdox as a zipped archive. (ZIP) [file pone.0065803.s004.zip › Skylark_ODdox/map__cfg_8h.html]

ALMaSS Skylark ODdox: map\_cfg.h File Reference


ALMaSS Skylark ODdox
 2.0


- Main Page
- Related Pages
- Classes
- Files

- File List
- File Members

Variables

map\_cfg.h File Reference

|  |  |
| --- | --- |
| Variables | |
| CfgBool | g\_farm\_enable\_crop\_rotation |
| CfgBool | g\_farm\_fixed\_crop\_enable |
| CfgInt | g\_farm\_fixed\_crop\_type |
| CfgBool | g\_farm\_fixed\_rotation\_enable |
| CfgBool | g\_farm\_fixed\_rotation\_farms\_async |
| CfgInt | g\_farm\_fixed\_rotation\_farmtype |
| CfgInt | g\_farm\_test\_crop\_daystorun |
| CfgInt | g\_farm\_test\_crop\_farmtype |
| CfgStr | g\_farm\_test\_crop\_filename |
| CfgBool | g\_farm\_test\_crop\_reportdaily |
| CfgInt | g\_farm\_test\_crop\_sample\_x |
| CfgInt | g\_farm\_test\_crop\_sample\_y |
| CfgInt | g\_farm\_test\_crop\_type |
| CfgInt | g\_map\_le\_border\_chance |
| CfgBool | g\_map\_le\_borders |
| CfgBool | g\_map\_le\_unsprayedmargins |
| CfgInt | g\_map\_le\_unsprayedmargins\_chance |
| CfgInt | g\_map\_maxpolyref |
| CfgBool | g\_map\_orchards\_borders |
| CfgInt | g\_map\_orchardsborderwidth |

---

## Variable Documentation

CfgBool g\_farm\_enable\_crop\_rotation

Referenced by Farm::GetNextCropIndex().

CfgBool g\_farm\_fixed\_crop\_enable

Referenced by Farm::GetNextCropStartDate(), Farm::HandleEvents(), and Farm::InitiateManagement().

CfgInt g\_farm\_fixed\_crop\_type

Referenced by Farm::HandleEvents(), and Farm::InitiateManagement().

CfgBool g\_farm\_fixed\_rotation\_enable

Referenced by FarmManager::CreateFarms(), and Farm::GetFirstCropIndex().

CfgBool g\_farm\_fixed\_rotation\_farms\_async

Referenced by Farm::GetFirstCropIndex().

CfgInt g\_farm\_fixed\_rotation\_farmtype

Referenced by FarmManager::CreateFarms().

CfgInt g\_farm\_test\_crop\_daystorun

CfgInt g\_farm\_test\_crop\_farmtype

CfgStr g\_farm\_test\_crop\_filename

CfgBool g\_farm\_test\_crop\_reportdaily

CfgInt g\_farm\_test\_crop\_sample\_x

CfgInt g\_farm\_test\_crop\_sample\_y

CfgInt g\_farm\_test\_crop\_type

CfgInt g\_map\_le\_border\_chance

Percentile chance of a field getting a border, if MAP\_LE\_BORDERS is enabled above.

Referenced by Landscape::ReadPolys().

CfgBool g\_map\_le\_borders

If true will make and use new borders around certain types of farmed areas. The area used is 'stolen' from a parent landscape element.

Referenced by Landscape::Landscape(), and Landscape::ReadPolys().

CfgBool g\_map\_le\_unsprayedmargins

Referenced by Landscape::Landscape(), and Landscape::ReadPolys().

CfgInt g\_map\_le\_unsprayedmargins\_chance

Referenced by Landscape::ReadPolys().

CfgInt g\_map\_maxpolyref

CfgBool g\_map\_orchards\_borders

If true will make and use new borders around tole\_orchard areas. The area used is 'stolen' from a parent landscape element.

Referenced by Landscape::Landscape().

CfgInt g\_map\_orchardsborderwidth

The width of the orchard borders if any

Referenced by Landscape::Landscape().


- CJT
- MSVC
- ALMaSS Working Source
- Landscape
- map\_cfg.h
- Generated on Thu Jan 10 2013 13:15:35 for ALMaSS Skylark ODdox by
   1.8.1.1
